# Editorial: Community series in mental illness, culture, and society: Dealing with the COVID-19 pandemic - Volume III

**DOI:** 10.3389/fpsyt.2023.1145115

**Published:** 2023-02-06

**Authors:** Renato de Filippis, Mohammadreza Shalbafan, Samer El Hayek

**Affiliations:** ^1^Psychiatry Unit, Department of Health Sciences, University Magna Graecia of Catanzaro, Catanzaro, Italy; ^2^Mental Health Research Center, Psychosocial Health Research Institute, Department of Psychiatry, School of Medicine, Iran University of Medical Sciences, Tehran, Iran; ^3^Medical Department, Erada Center for Treatment and Rehab in Dubai, Dubai, United Arab Emirates

**Keywords:** coronavirus, lockdown, mental health, mental disorders, psychiatry, psychological impact, social distancing, social isolation

Three years after the global coronavirus pandemic outbreak, its severe and manifold consequences have been largely demonstrated not only in terms of global economy, culture, society, and general health, but also mental health (1–3). Many aspects of daily life have been affected by the lockdown and social distancing measures, further filtered by individual factors, as well as specific sociocultural dynamics, including norms, values, and religions (4–8).

Continuing the path taken in the previous two volumes of the Community Series of our Research Topic entitled “*Mental Illness, Culture, and Society: Dealing with the COVID-19 Pandemic”* (9, 10), this third Volume collects nine new papers exploring, in different ways, the complex relationship between the COVID-19 pandemic and mental health, mediated in distinctive ways by peculiar cultures, societies, and backgrounds all over the world.

Five studies dealt with the differential impact of COVID-19 on mental health-related symptoms in special populations. The team lead by Macaron et al. conducted a systematic review and meta-analysis to explore geographical differences of physicians' burnout during the early and late phases of the pandemic. The synthesis of 45 cross-sectional studies demonstrated a 54.60% overall prevalence of burnout (from 60.7% in the early phase to 49.3% in the late pandemic period), particularly marked among frontliners and those residing in the Middle East and North Africa region, and mainly characterized by emotional exhaustion, depersonalization, and personal accomplishment.

The paper published by Nishat et al. assessed the mental health burden of 304 early married girls in Bangladesh during the pandemic. They found a depression and anxiety rates of 60.9 and 23.7%, respectively, with higher peaks in younger girls and those belonging to the Sanatan (Hindu) religion compared to older and Muslim participants.

Heesen et al. presented results from their longitudinal observational study over three time points, evaluating the role of COVID-19 vaccination on social participation and mental health status in immunocompromised individuals. Overall, 60% of the 126 participants increased their social participation over time after the vaccination. The authors did not find any association between social participation and mental health, sociodemographic, or medical factors except hypertension.

The paper by Wegner and Liu focused on the differences in positive and negative experiences of lonely and non-lonely people during the COVID-19 pandemic in Germany. According to this cross-sectional online survey involving 1,758 participants, lonely individuals reported fewer positive experiences and more negatives ones of living in the COVID-19 pandemic, as compared to non-lonely individuals. Interestingly, compared to non-lonely participant, lonely people were less likely to view the pandemic as a conspiracy.

The last paper addressing a special population came from Ransing et al. who developed a stepped-care model for perinatal women, that aims to tackle COVID-19 vaccine hesitancy (CVH) in low- and middle-income countries (LMICs). After a systematic review of the literature, the team, consisting of vaccinators, experts, and stakeholders, reached a consensus about a COVID-19 Vaccine Confidence Project for Perinatal Women (CCPP). The CCPP model included health care personnel training, integration into ongoing COVID-19 vaccination programs, CVH screening, CVH intervention, and referral services suitable for implementation in LMICs.

On the other hand, three included papers used peculiar study design to add some elements of novelty to the field. Hajebi et al. proposed a study protocol of a randomized controlled trial aiming to explore the efficacy of three vs. five sessions of grief counseling on the psychological aspects following COVID-19 bereavement. This multi-center study will enroll 120 people in bereavement due to COVID-19 in either three-session or five-session grief counseling intervention groups; the trial will span over 3 months.

Wang et al. proposed a mixed methods research study to examine short message service (SMS) as a way of meeting the public's need for emotional support during the COVID-19 pandemic in China. According to the authors' findings, SMS messages had some efficacy only in the elderly population while, with the stabilization of COVID-19, SMS has once again been discarded by users, especially digital natives.

Fu et al. presented a visual analysis using co-occurrence and VOSviewer bibliometric methods to analyze the evolution of research on depression during COVID-19. The authors demonstrated a global breakthrough unprecedented in the history of research, with universities in the United States, China, and the United Kingdom having the largest number of publications, and in a close cooperation with each other.

Through a cross-sectional population-based study conducted in South Korea, Lee et al. assessed the socio-economic factors associated with mental health outcomes during the COVID-19 pandemic. According to the authors' conclusions, the most affecting risk factors for depressive and anxiety symptoms were being single, reporting a lower household income, lower support from friends or family, and increased stress from the workplace or home.

Summarizing, the articles collected in this third Volume of our Research Topic continue to emphasize the role of different cultural and social features in the responses and psychiatric consequences to the COVID-19 pandemic throughout the world. While, on the one hand, the immediate impact of the pandemic has proved to be above all economic and medical in general, the medium- to long-term effects are substantial on mental health and remain still far from being resolved.

## Author contributions

All authors listed have made a substantial, direct, and intellectual contribution to the work and approved it for publication.
